# Dual Coating of Liposomes as Encapsulating Matrix of Antimicrobial Peptides: Development and Characterization

**DOI:** 10.3389/fchem.2017.00103

**Published:** 2017-11-17

**Authors:** Ahmed I. Gomaa, Cynthia Martinent, Riadh Hammami, Ismail Fliss, Muriel Subirade

**Affiliations:** ^1^Department of Food Science, Laval University, Quebec City, QC, Canada; ^2^Institute of Nutrition and Functional Foods, Quebec City, QC, Canada; ^3^National Research Center, Food Science and Nutrition Department, Cairo, Egypt; ^4^Faculty of Health Sciences, School of Nutrition Sciences, University of Ottawa, Ottawa, ON, Canada

**Keywords:** coated liposomes, encapsulation, antimicrobial peptides, bacteriocins, dissolution

## Abstract

Antimicrobial peptides have been proposed as a potential biopreservatives in pharmaceutical research and agribusiness. However, many limitations hinder their utilization, such as their vulnerability to proteolytic digestion and their potential interaction with other food ingredients in complex food systems. One approach to overcome such problems is developing formulations entrapping and thereby protecting the antimicrobial peptides. Liposome encapsulation is a strategy that could be implemented to combine protection of the antimicrobial activity of the peptides from proteolytic enzymes and the controlled release of the encapsulated active ingredients. The objective of this study was to develop dual-coated food grade liposome formulations for oral administration of bacteriocins. The formulations were developed from anionic and cationic phospholipids as models of negatively and positively charged liposomes, respectively. Liposomes were prepared by the hydration of lipid films. Subsequently, the liposomes were coated with two layers comprising a biopolymer network (pectin) and whey proteins (WPI) in order to further improve their stability and enable the gradual release of the developed liposomes. Liposomes were characterized for their size, charge, molecular structure, morphology, encapsulation efficiency, and release. The results of FTIR, zeta potential, size distribution, and transmission electron microscopy (TEM) confirmed that the liposomes were efficiently coated. Ionic interactions were involved in the stabilization of the positively charged liposome formulations. Negatively charge liposome formulations were stabilized through weak interactions. The release study proved the efficiency of dual coating on the protection of liposomes against gastrointestinal digestion. This work is the first to study the encapsulation of antimicrobial peptides in dual-coated liposomes. Furthermore, the work successfully encapsulated MccJ25 in both negative and positive liposome models.

## Introduction

The accelerating spread of antibiotic resistance by pathogenic bacteria has become a major public health problem. Accordingly, there is increasing demand for natural antimicrobial components with innovative modes of action. Antimicrobial peptides have been proposed as potential biopreservatives in pharmaceutical research and agribusiness to replace chemical preservatives. Among bacteriocins, microcin J25 (MccJ25), produced by Gram-negative bacteria, has potent bactericidal activity against a range of pathogenic enteric bacteria such as *Escherichia coli* and *Salmonella* with a specific mechanism of action (Hammami et al., 2015).

A production technique for MccJ25 has been developed, including rapid two-step purification, that allowed the application of this molecule in different fields (food, medical, and veterinary) where the need for such new molecules is increasingly urgent (Sable et al., 2000). However, many limitations hinder the utilization of antibacterial peptides including their vulnerability to proteolytic digestion and their potential interaction with other food ingredients in complex food systems. One key approach to overcoming such problems is by developing formulations entrapping the designed antimicrobial peptides (Narsaiah et al., 2013). Various forms of encapsulation such as liposomes, films or beads, may be used to achieve controlled release of antimicrobial peptides (Millette et al., 2007; da Silva Malheiros et al., 2010), where encapsulation enables maintenance of antimicrobial activity and stability of active ingredients in complex systems. Encapsulation in liposomes is a strategy that could be implemented to provide stability to antimicrobial peptides, protect their antimicrobial activity, and to control the release of the encapsulated active ingredients (da Silva Malheiros et al., 2010).

Liposomes are self-assembled colloidal systems of vesicles composed of one or more phospholipid bilayers, which resemble the cell. Liposomes are a promising delivery system for encapsulation and delivery of water soluble molecules because of their protective ability, encapsulation capacity, and biocompatibility (Lasic and Papahadjopoulos, 1995). The mechanism of liposome formation is based on the dual hydrophobic and hydrophilic properties of phospholipids, whereby interactions occur between polar head-groups of phospholipids and the aqueous phases, while the hydrophobic hydrocarbon tails form a bilayer facing each other (Jesorka and Orwar, 2008). Previous research has shown that liposome encapsulation provides numerous benefits to encapsulated materials such as protection against enzymes and buffering against pH changes (da Silva Malheiros et al., 2010). Liposomes have the advantages of large carrying capacity of hydrophilic or hydrophobic substances. However, practical applications of liposomes in the food industry are limited because of their poor physical and digestive stability during passage through the gastrointestinal tract due to the sensitivity of liposomes to proteases. Also, environmental conditions such as pH and temperature can cause structural damage to the liposomes. In addition, the bile salts accelerate the hydrolysis of the lipid bilayer by increasing the fluidity of the membrane (Liu et al., 2015) and the low pH such as under gastric conditions cause changes in the surface charge of the liposomes (Lähdesmäki et al., 2010).

The stability of the liposomes to gastrointestinal conditions can be increased by using coatings of materials such as chitosan, alginate, pectin or whey protein (Nguyen et al., 2011; Liu et al., 2013; Frenzel and Steffen-Heins, 2015; Frenzel et al., 2015). Researchers have recently coated pectin/alginate microparticles with whey protein isolate (WPI) due to the ionic interactions between oppositely charged polysaccharides and proteins (Aguilar et al., 2015). The muco-adhesive properties of liposomes were improved by coating with pectin (Nguyen et al., 2011; Klemetsrud et al., 2013). WPI has been also used as an encapsulating matrix for active ingredients such as peptides. In addition, WPI is used in many food applications because of its multiple functional properties and GRAS status (Aguilar et al., 2015). Recently, it has been shown that the use of WPI as a coating agent for liposomes increased their stability due to its ability to decrease their semi-permeability and resistance to osmotic forces that exist in food matrices with a high concentration of sugar or salt (Frenzel et al., 2015). Thus, the WPI and pectin were proved to be two very interesting materials for improving the encapsulation and release of antimicrobial peptides. However, very limited studies have been performed on improving the effectiveness of liposomes encapsulation by coating materials. The objective of this study was to develop a food-grade, dual-coated liposomes encapsulating the antibacterial peptide (MccJ25) to enable (provide) protection against gastrointestinal digestion and improve its controlled release.

## Materials and methods

### Production and purification of MccJ25

Bacteriocin MccJ25 was produced by *E. coli* pTUC202 strain in a minimal condition using M63 medium (KH_2_ PO_4_ (3 g/L), K_2_HPO_4_ (7 g/L), (NH_4)_ H_2_PO_4_ (2 g/L), casamino acids (1 g/L) supplemented with glucose, MgSO_4_ and thiamine). The M63 medium was inoculated with 1% of the active medium in culture strain (Luria Bertani medium supplemented with chloramphenicol) and incubated at 37°C for 18 h with stirring. The supernatant free of cell culture was collected by centrifugation at 8,000 g for 20 min at room temperature. Thereafter, the supernatant was initially purified on a Solid Phase Extraction (SPE) using Sep-Pak C18 column (low pressure chromatography) at 4°C using different fractions of acetonitrile and a flow rate of 10 mL/min to separate the molecules according to their hydrophobicity. The antimicrobial activity of the various fractions was determined by an agar diffusion test. The fraction that showed activity was frozen and then lyophilized. Subsequently a second purification step was performed by a preparative HPLC and the microbial activity was measured. The purified MccJ25 was stored lyophilized until encapsulation in liposomes.

### Protein determination

During the purification steps, the protein concentration of solutions containing the MccJ25 was determined by the Lowry method (Lowry et al., 1951) using the absorbance at 750 nm and a standard curve prepared from bovine serum albumin with a coefficient of determination *r*^2^ of 0.99.

### Measuring the activity of MccJ25

Two different methods namely, agar diffusion, and critical dilution method, were used to measure the activity of MccJ25.

### Agar diffusion test

The purified bacteriocin was tested for antimicrobial activity against *Salmonella enteritidis* (National Museum of Natural History, Washington, DC) with a MIC of 0.04 mM as previously described (Hammami et al., 2009). The strain was inoculated at 1% in a soft LB medium at 45°C, supplemented with 0.75% agar. Inoculated agar was poured into sterile Petri plates and allowed to solidify for 10–15 min in a biological hood. Once the agar solidified, wells were bored using the wide end of a 5 ml sterile pipette, then 80 μl of each fraction was dispensed into the wells and incubated 18 h at 37°C. After incubation, the activity was determined by measuring the diameter of the inhibition zone.

### Microtitration assay

The second activity test was carried out using the same strain (*S. enteritidis*) in polystyrene 96-well micro-assay plates (Microtest, Becton–Dickinson Labware, MD, USA) as described by Ennaas et al. (2016). Briefly, fractions were added to microplates and diluted 2-folds with LB broth. Then, wells were seeded with ~1 × 10^4^ CFU of strain per well using log-phase culture diluted in LB to 0.5–1.0 × 10^6^ CFU ml^−1^. The plates were incubated for 18 h at 37°C. After incubation, OD at 595 nm was measured using an Infinite® F200 PRO photometer (Tecan US Inc., Durham, NC). Antimicrobial activity results were expressed as arbitrary units per milliliter (AU ml^−1^) that calculated as follows:

AU ml^−1^ = (1,000/100) × 2 n, where *n* = number of inhibited wells.

### Production of liposomes

The liposomes were made from 1,2-dimyristoyl *sn*-glycero-3-phosphocholine (DMPC), 1,2-dimyristoyl-sn-glycero-3-phospho-rac-(1-glycerol) with salt sodium (DMPG) and 1,2-dipalmitoyl-3-trimethylammonium propane (DMTAP). All phospholipids were food-grade purchased from Avanti Polar Lipids, Inc. (Alabaster, AL). Liposomes were prepared by lipid film hydration methodology followed by extrusion as described previously (Klemetsrud et al., 2013).

Anionic liposomes were prepared from a mixture of DMPC / DMPG (at a molar ratio of 10:1, respectively), while cationic liposomes were prepared from a mixture of DMPC / DMTAP at a molar ratio of 10:1, respectively. The phospholipids were dissolved with a chloroform /methanol solution (2:1 v/v) in a round bottomed flask. The final concentration of lipids was 6 mM. Then, the solvent was evaporated completely on a rotary evaporator (at *T* = 50°C). The resulting lipid film was placed under vacuum for 8 h to remove any residual solvent. Thereafter, the vesicles were obtained by hydration of the lipid film (at *T* = 30°C) in a buffer solution of 5 mM phosphate pH 7 for 2 h. Similarly, loaded liposomes were prepared by adding MccJ25 to the phosphate buffer at a concentration of 1 mg/mL. Liposomes were homogenized by four cycles of extrusion through a polycarbonate filter 1 μm using Lipex extruder (Northern, Lipids Inc., Vancouver, BC) with a double-walled chamber of 1.5 mL followed by another four cycles extrusion through a polycarbonate filter of 200 nm. Lipid solutions were extruded above the phase transition temperature (*T*_m_) of 24°C. The liposomes thus obtained were stored at 4°C.

### Liposomes coating

The liposomes were then coated by the method of ‘layer by layer’ with a layer of WPI or pectin and subsequently coated with another layer of the other component of WPI or pectin. Prior to the coating, pectin was purified in three steps to remove the possible degraded pectin, aggregates or salt, and to obtain a fine distribution of molecular weight. The first step was the centrifugation of an amidated pectin solution, 1.5% w/w (Sigma-Aldrich, St. Louis, Missouri) for 2 h at 20°C. The centrifugation was repeated twice after each centrifugation the supernatant was recovered. Secondly, the recovered solutions were dialyzed against distilled water at 4°C using 12–14 kDa molecular weight cut-off dialysis tubing for 7 days. The water was changed daily. Finally, the recovered pectin solution was lyophilized.

For coating the liposomes with a pectin layer, the purified pectin was dissolved at a concentration of 0.2% (w/w) in 5 mM phosphate buffer, pH 7, under magnetic stirring at room temperature overnight. To minimize the risk of particles of dust and contaminants, the pectin solution was filtered through a polycarbonate membrane of 1 mm at 45°C with a Lipex extruder. The liposome solution was added dropwise to the pectin or whey protein solution with a ratio of 1: 3 (v/v), respectively, under magnetic stirring at a rate of 2 ml/min and the samples were then stirred for an additional of 5 min and left overnight at 4°C.

The coating layer with WPI was performed using WPI (97% purity, BiPRO WPI, Davisco Foods International, Le Sueur, MN) dissolved in 5 mM phosphate buffer at different concentrations of 0.1, 0.6, and 3% (w/w). These above mentioned concentrations were used for the optimization of the coating layer with the WPI. Based on the results, only the concentration of 3% was used for the rest of the study. To study the effect of pH on the coating process of anionic liposomes, the buffer was pre-adjusted to pH 4.5 or pH 7, while for cationic liposome only adjusted buffer at pH 7 was used. After the first layer of coating, to remove the residuals of pectin or WPI, coated liposomes were centrifuged at 10,000 g for 30 min at 4°C. After the centrifugation the supernatant was removed and the pellet was similarly resuspended in the other solution of WPI or pectin for the second coating layer. Coated liposomes were centrifuged at 10,000 g for 30 min at 4°C. After the centrifugation the supernatant was removed and the pellet was resuspended in the same volume of the buffer. The coated and purified liposomes were then stored in a refrigerator at 4°C and were used within 2 week time interval. All coating steps were performed at 4°C.

### Physicochemical characterization of liposomes

#### Particle size distribution and zeta potential

The particle size distribution of liposomes was studied by laser diffraction using the dynamic light scattering method using Zetasizer Nano-ZS (Malvern Instruments, Southborough, MA). The samples were diluted 10-fold with distilled water and stirred for 1 min, then were introduced into a semi-micro quartz cuvette. The measurements were started after 2 min of equilibration of the cell temperature at 20°C. Measurements were performed using the red laser with a refractive index of 1.44 and absorption value will be 0.01. Data analysis was performed by intensity.

To measure the surface charges of liposomes, zeta potential measurements (electrophoretic mobility) of 10-fold diluted samples were performed at 4°C using an aqueous immersion cell in the automatic mode.

#### Molecular structure characterization

The analysis of molecular structure was performed by FTIR spectroscopy in the transmission mode. Samples were initially lyophilized. Then, 1 mg of sample was ground and homogenized with 100 mg of KBr previously dried overnight in an oven at 50°C. The mixture was then pressed into a tablet for about 10 s using a hydraulic press. The infrared spectra of the KBr tablets were recorded using a Nicolet 6700 spectrometer (Madison, WI) equipped with a deuterated triglycine sulfate detector (DTGS) continuously purged with dry air.

Before each measurement, the chamber of the spectrometer sample was purged with dry air for 15 min. The spectra were recorded in the mid-infrared range (400–4,000 cm^−1^). For each spectrum, 512 interferograms were co-added at 4 cm^−1^ resolution and Fourier transformed employing Happ-Genzel apodization. Spectra were baselines corrected and normalized as previously described (Gomaa et al., 2013).

#### Morphological study

The transmission electron microscopy (TEM) was used to observe the internal morphology of liposomes. Samples are fixed using 2.5% glutaraldehyde and mounted on metal grids. Staining was performed using uranyl acetate for 1 min and then the samples were rinsed by immersion in deionized water and dried with filter paper. Observations were made at high resolution (80 kV) with a JEOL 1200EX electron microscope (JEOL Ltd., Akishima, Japan).

#### Encapsulation efficiency

Direct HPLC method was used to measure the MccJ25 encapsulated liposomes. The samples were analyzed with a HPLC system (Agilent HP series 1,100) equipped with an analytical C18 reverse phase column (250 × 4.6 mm Phenomenex Luna Å) at 40°C and a diode array detector (DAD) at 214.8 nm wavelength. Solvent A (1L of HPLC water acidified with 416 μL HCl) and Solvent B (HPLC-grade acetonitrile). The samples (100 μL) were injected and eluted at a flow rate of 1.5 mL/min with a linear gradient of solvent B from 0 to 40% in 40 min. The encapsulation efficiency (% EE) was measured by comparing the quantities of MccJ25 before and after encapsulation.

#### *In vitro* digestion model

The dissolution experiment was performed according to Liu et al. (2012) with some modifications. Different samples were placed in a simulated gastric fluid for 2 h (SGF: 0.2 g of NaCl dissolved in 0.7 mL of HCl and filled up to 100 ml with distilled water then pepsin was added at a concentration of 3.2 mg/mL). Then, the dissolution was performed in simulated intestinal fluid (SIF: 6.8 g of potassium phosphate monobasic sodium hydroxide with a pH adjusted to 7.4 with 2N sodium hydroxide to a volume of 100 mL and a concentration of 0.2 mg/mL bovine bile extract), the pH was adjusted to 7.4. Pancreatin was added at a concentration of 20 mg/mL (equivalent to a protease activity of 500 U/mL). The dissolution was performed for 4 h at a temperature of 37 ± 0.5°C with stirring at 140 rpm. To perform the experiment 3 ml of the individual suspensions of liposomes was added to 3 mL of gastric fluid and samples of 500 μL of medium are removed at intervals of 1 h for the gastric fluid portion and at 0, 15, 30, 60, 120, 240 min during the intestinal dissolution. The contents MccJ25 samples were determined by HPLC. Because of the thermal stability of MccJ25, the samples were heated at 100°C for 5 min to inactivate the digestive enzymes. The blank was performed in similar conditions with a solution of non-encapsulated MccJ25.

### Statistical analysis

Statistical analysis was performed using SAS software for Windows (Version 9.4) followed by one-way an analysis of variance (ANOVA). Comparisons of means were performed using the Duncan's method with a significance level *p* < 0.05. The measurements were made in duplicate and the results were reported as mean ± standard deviation.

## Results and discussion

### Production of the microcin

**Figure 1:**
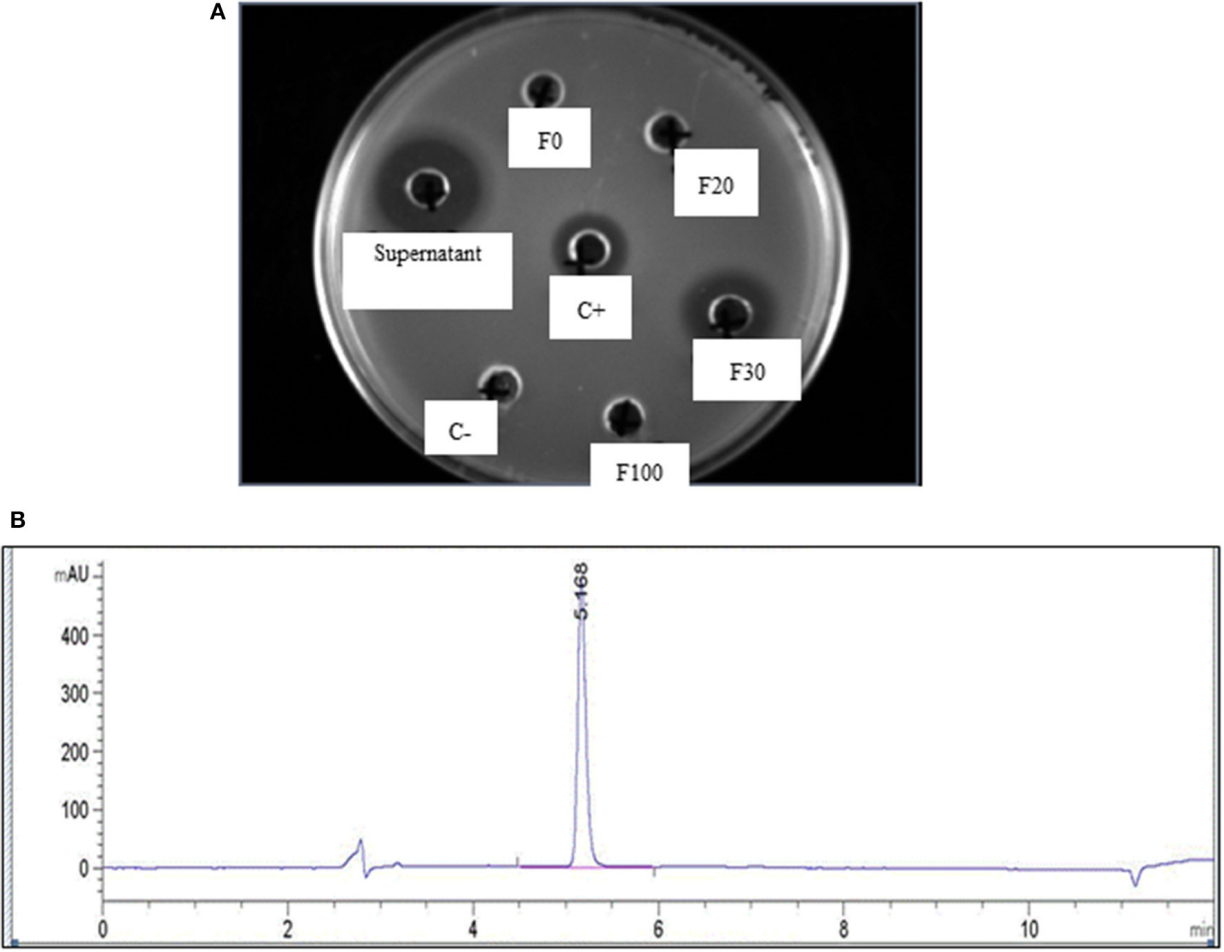
**(A)** Activity of different fractions obtained from initial purification by Sep-back Solid Phase Extraction (SPE) as measured by agar diffusion test using different fractions of acetonitrile and a flow rate of 10 mL/min; F0, F20, F30, and F100 are the fractions obtained at 0, 20, 30, and 100% Acetonitril, respectively; C+ and C– are positive and negative control, respectively; **(B)** HPLC chromatogram of purified MccJ25 after purification using the preparative HPLC.

The production of MccJ25 was monitored by determining the protein concentration and bactericidal activity. After the purification steps, 43 mg of purified MccJ25 was obtained from 2.25 L of supernatant of *E. coli* MC4100 PTUC 202 (Table 1). Antimicrobial activity tests were used to determine the activity of the fractions obtained from Sep-Pak SPE and the inhibitory activity of the resulting peptide. The fraction containing 30% ACN was the fraction containing the active peptide after the purification step with Sep-Pak (Figure 1). The purification steps increased the protein concentration from 0.23 to 2.89 mg/mL and the specific inhibitory activity was also increased from 35,396 to 181,352 AU/mg with a purification coefficient of 5.12 times. During the current study, 80 mg of MccJ25 were utilized (produced from two batches).

**Table 1:** Production and purification steps of microcin J25.

**Sample**	**Volume (mL)**	**protein concentration (mg/ml)**	**Quantity of protein (mg)**	**Inhibitory activity (AU/ml)**	**Total activity (AU)**	**Specific activity (AU/mg)**
Supernatant	2,250	0.23 ± 0.04	520.73	8,192	18,432,000	35,396
After Sep-Pak	193	0.31 ± 0.17	60.37	32,768	6,324,224	104,758
After HPLC	15	2.89 ± 0.88	43.36	524,288	7,864,320	181,352

*Protein content were measured by Lowry method, inhibitory activities were measured by the microtitration assay. Results are expressed as average ± standard deviation*.

### Physical properties of different liposomal formulations

**Figure 2:**
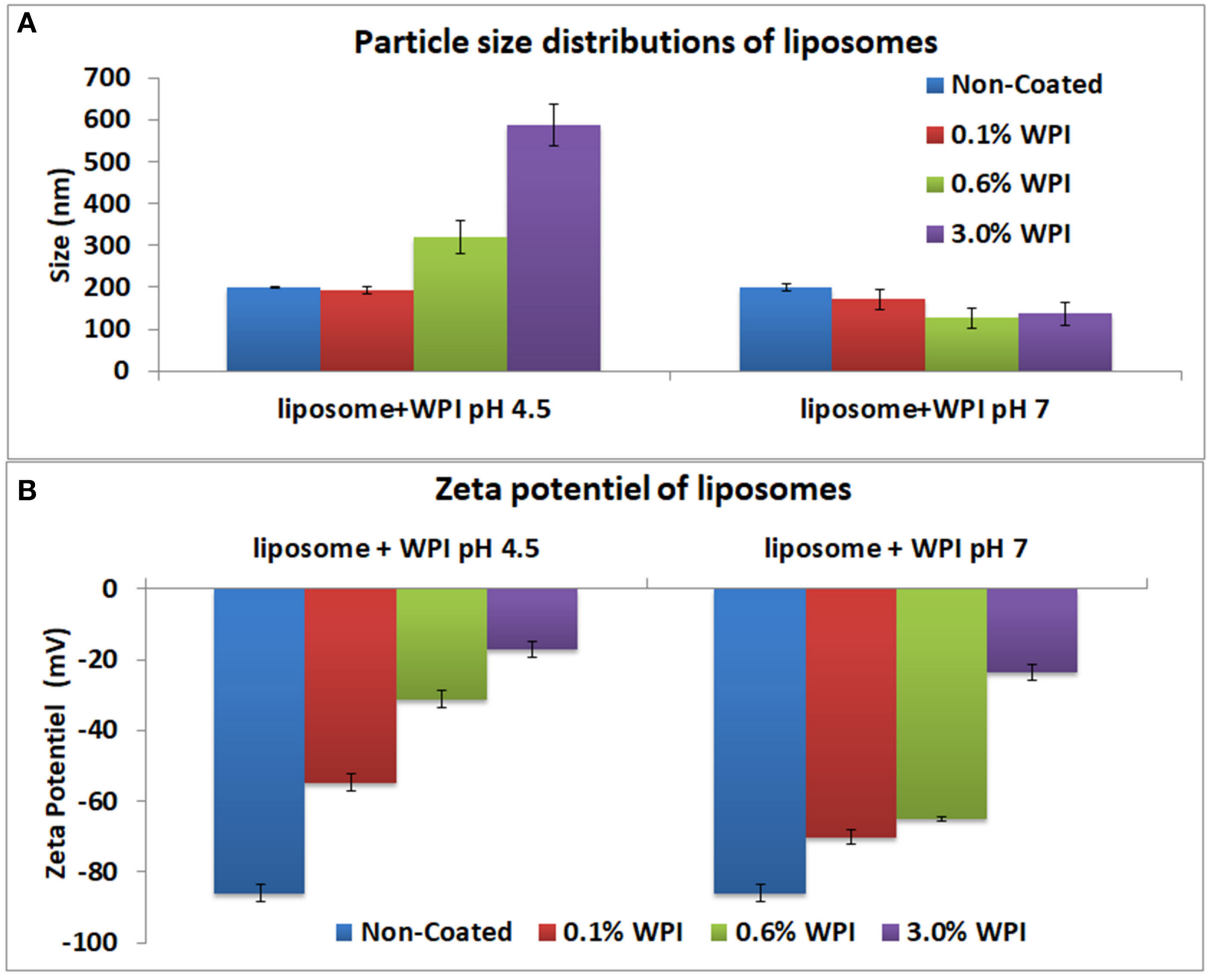
Particle size distributions **(A)** and of the zeta potential **(B)** of the liposomes at 0.1, 0.6, and 3% WPI concentration as measured by laser diffraction methodology. Error bars indicate the standard deviation of two measurements.

The analysis of the particle size distributions of liposomes did not show any aggregates. The polydispersion index ranged from 0.05 to 0.2. Uncoated negative liposomes showed a mean diameter of 199 ± 2 nm and a negative zeta potential (ζ-potential) of −85 ± 2 mV (Figure 2).

### Whey optimal concentration

The optimal concentration and pH of WPI solutions for sufficient liposomes surface coverage was determined using negative liposomes. Three concentrations of WPI were investigated: 0.1, 0.6, and 3% (w/w) at pH 4.5 and 7. The zeta potential and size of WPI were 1.98 ± 0.2 mV and 12.97 ± 0.4 nm, respectively). At pH 4.5, no change was observed in the size with the addition of 0.1% WPI, however, the zeta potential was increased to −55 mV. For 0.6 and 3% WPI, the size of the liposomes increased to 320 and 588 nm accompanied by a significant increase in the zeta potential to −31 and −17 mV, respectively. The results indicated that the concentrations of 0.1 and 0.6% WPI were inadequate to cover the entire surface of the liposome. The zeta potential of liposomes coated with 3% WPI reached a high level of neutralization of the surface of liposomes. Higher WPI concentrations did not increase the size or the zeta potential of the liposomes (results not shown). The zeta potential of whey covered liposomes did not reach a complete neutralization of the liposomes' negative charge, which indicated that WPI did not form a continuous layer covering the liposomes or alternately, WPI interacted and penetrated the liposomes. As such, the interaction between liposomes and whey protein could be explained by the insertion of the protein into the membrane which is in agreement with (Frenzel and Steffen-Heins, 2015). At pH 4.5, the charge of WPI is generally positive as the pH is below the isoelectric point of β-lactoglobulin (pI = 5.2), the major protein in WPI. Therefore, liposome coating could be explained by electrostatic interactions between the positive charge of the whey and the negative charge of the liposomes. At pH 7, no significant change in the liposome size was observed, indicating inefficient coating due to repulsions between the negative charges of whey and liposomes. Thus, the optimal coating with WPI was obtained with a 3% concentration at pH 4.5. As such, that concentration was used in the further experiments.

### Coating the anionic liposomes

**Figure 3:**
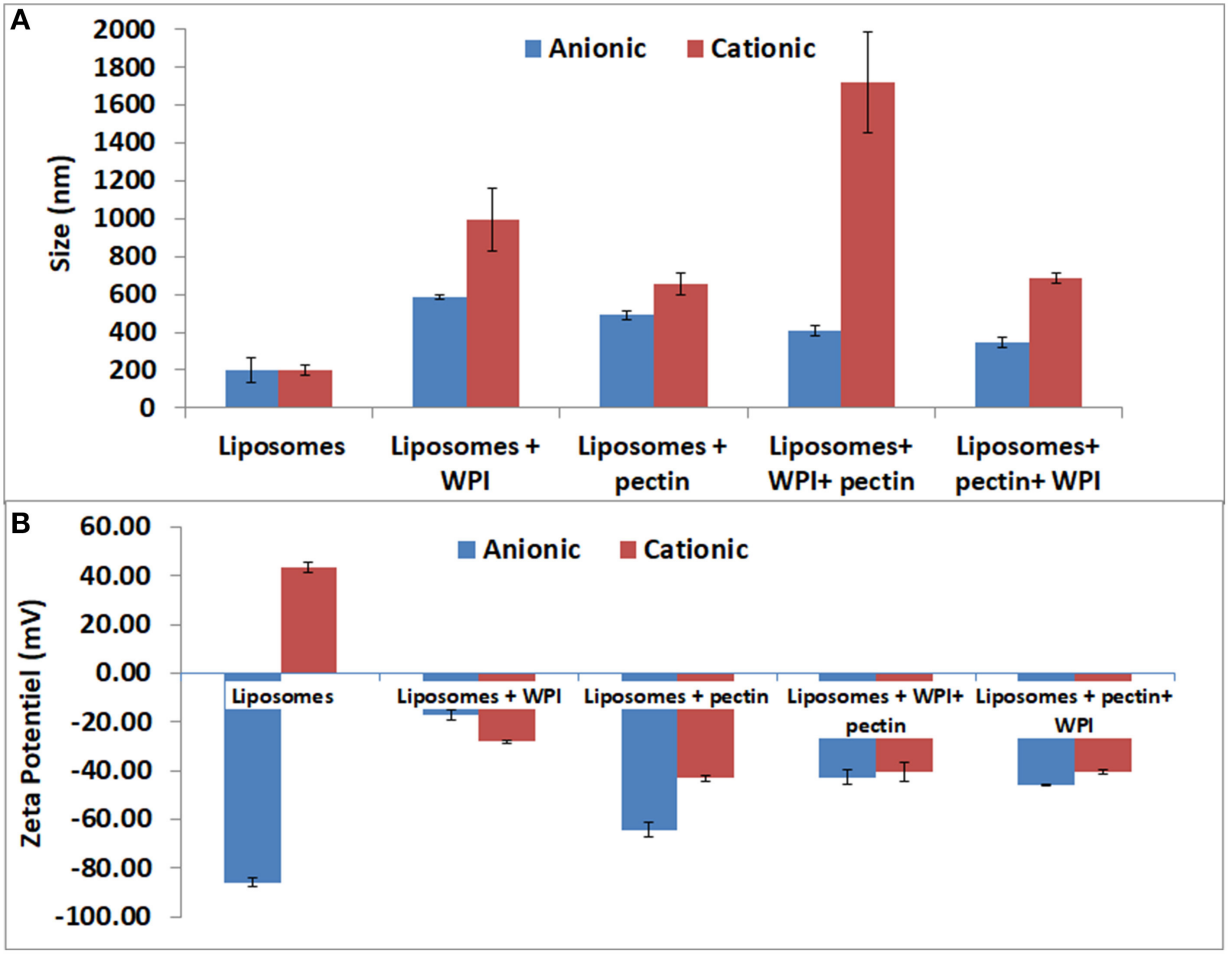
Particle size distributions **(A)** and of the zeta potential **(B)** of positive and negative liposomes coated with WPI and /or pectin as measured by laser diffraction methodology. Error bars indicate the standard deviation of two measurements.

The size and the zeta potential of the anionic and cationic liposomes after coating with whey and or/pectin are presented in Figure 3. The ζ-potential of liposomes coated with WPI and subsequently coated with pectin were similar to that of pectin (ζ-potential = −43 ± 0.7 mV confirming complete coverage of the liposome surface by a pectin layer through the interaction between pectin and whey. Moreover, pectin coating significantly decreased the particle size of liposome-coated whey. Decreasing the size of the liposomes can be explained by a compression effect of whey protein particles, after their interaction with pectin, on liposomes. These results were consistent with the findings of Frenzel et al. (2015). In this study, non-denatured WPI was utilized similar to previous literature that observed a higher adsorption for pectin particles coated with non-denatured whey protein compared with thermally denatured whey (49.2 vs. 27.6%, respectively) when 4% of proteins in solution were used (Souza et al., 2012).

Furthermore, the coating of liposomes by a negative layer of pectin (as a first layer) caused a significant increase in liposome size from 199 to 490 nm and the zeta potential was close to that of the pectin. The results confirm the possibility of coating negatively charged liposomes with negative polymers such as pectin. The results are in agreement with those obtained for liposomes coated with different forms of pectin (Nguyen et al., 2011). Efficient coating of anionic liposomes and pectin may be explained by the interactions between amide groups of pectin and liposomes, which needs to be verified with further analysis (FT-IR and TEM) because there is no possibility of electrostatic interactions as pectin and anionic liposomes have the same charge. The second coating step by WPI, after coating with pectin, involved a reduction in size to 344 nm while the zeta potential was increased from −64 to −46 mV. The size reduction could be explained by the compression effect driven by the WPI (Souza et al., 2012; Frenzel and Steffen-Heins, 2015).

### Coating the cationic liposomes

The data on the size and the zeta potential of the cationic coated liposomes are presented in Figure 3. Coating cationic liposomes with WPI resulted in an increased size to 997 nm accompanied by a change in the zeta potential from 47 to −28 mV. Indeed, at pH 7 the WPI is generally negatively charged which may explain the electrostatic interaction with the charge of the liposome. The second coating layer by pectin led to an increase in particle size to 1,720 nm accompanied with a change in zeta potential to −40 mV. This can be explained by the fact that although the WPI is negatively charged, it does not completely neutralize the positive charge of the liposome on which the pectin may be adsorbed subsequently, as reflected by the increase in the size and a change in the zeta potential close to that of the pectin.

Liposomes coating with a first layer of pectin resulted in significant increase of the particle size to about 300 nm which is in agreement with Nguyen et al. (2011) who coated positive liposomes with a pectin layer of about 400 nm. The increase in size was accompanied by a significant decrease in zeta potential to negative a value close to that of pectin. These results confirmed the effectiveness of pectin coating. The effect of pectin coating could be explained by the ionic interaction between the positive liposome charges and negative charges of pectin. Subsequent coating by WPI, after coating with pectin, did not result in significant increase in the particle size or in the zeta potential. Accordingly, these findings could indicate that the pectin completely covered the surface of liposomes thus neutralizing their charge and created a steric hindrance that prevented the whey protein from subsequently adsorption. However, a recent study (Souza et al., 2012) observed a decrease in the size of pectin particles during the coating with WPI that was attributed to the pressing effect of WPI which caused the expulsion of part of the water present in the particles. FT-IR and microscopy analysis were performed to complement and confirm the results.

### Molecular structure analysis

**Figure 4:**
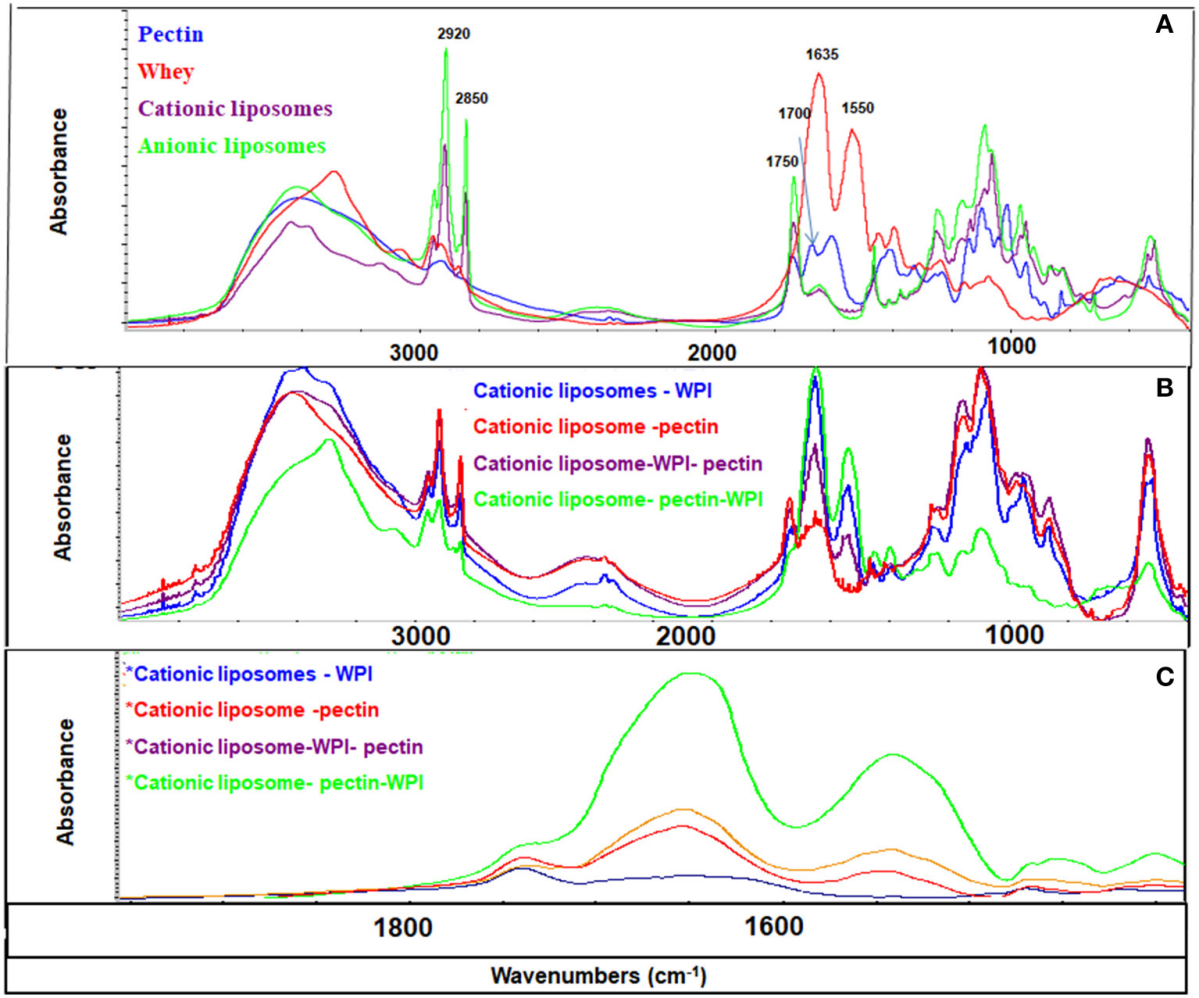
FT-IR spectra of liposomes and coating materials (WPI and pectin) **(A)**; cationic liposomal formulas **(B)**; and 1,900–1,400 cm^−1^ area **(C)**. The peaks at 2,850 and 2,920 cm^−1^ represent symmetric and asymmetric stretching vibrations of CH_2_ groups, the peak at 1,750 cm^−1^ represents the ester group of the phospholipids, the peak at 1,635 cm^−1^ represents C = O stretching vibration of amide I band, the peak at 1520–1580 cm^−1^ represents the amide II band, and the peak at 1,700 cm^−1^ represents the amidation group of the pectin.

**Figure 5:**
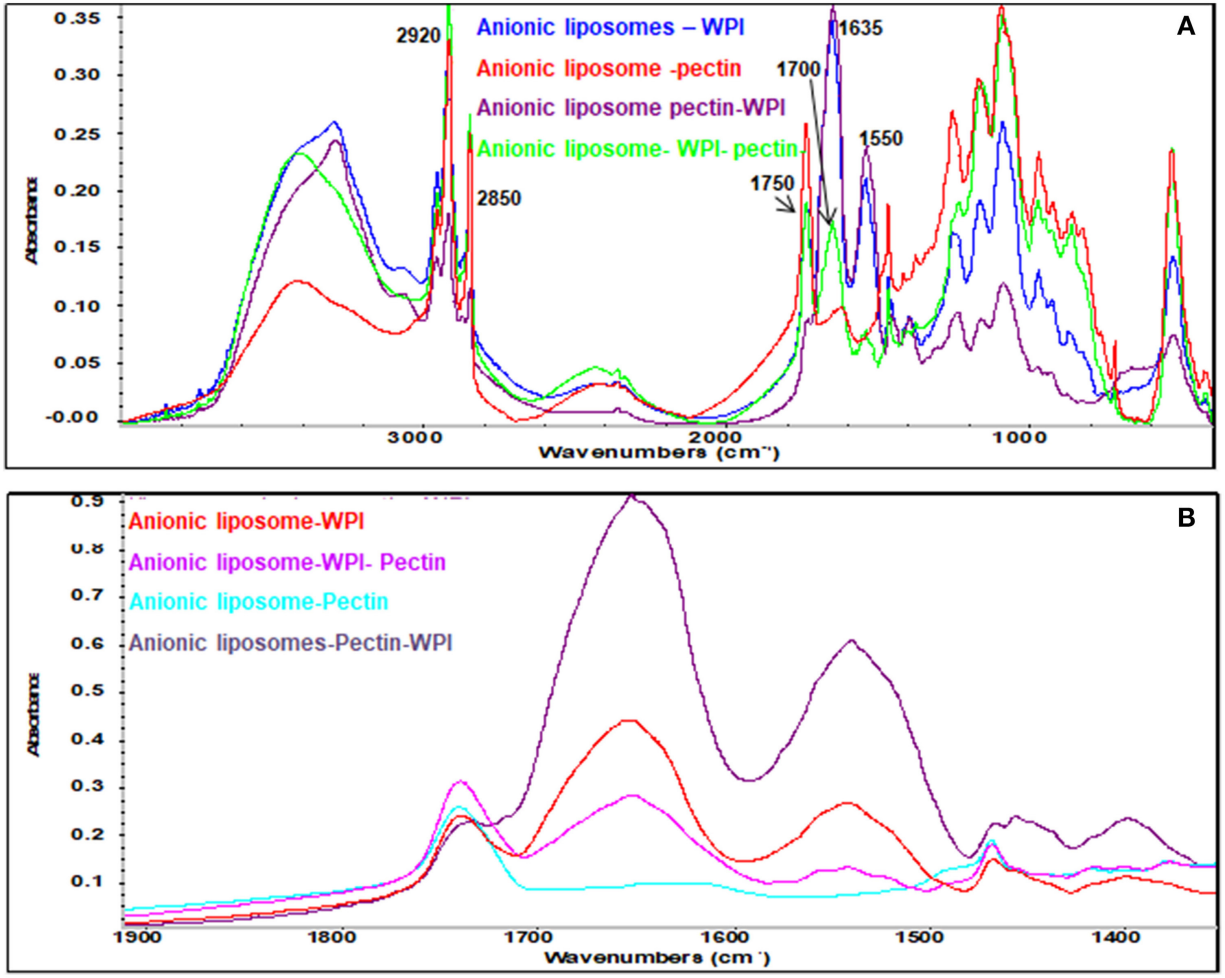
FT-IR spectra of anionic liposomal formulas: **(A)** Mid-IR range and **(B)** 1,900–1,400 cm^−1^ range.

Efficiency of coating could be monitored and confirmed by FTIR. Functional groups of each component give rise to characteristic bands in the mid-IR region. For liposomes, two characteristic bands corresponding to the symmetric and asymmetric stretching vibrations of CH2 groups are located at 2,850 and 2,920 cm^−1^, respectively. These bands provide conformational information of the lipid acyl chains existing in all liposome formulations (Figures 4, 5). WPI is characterized by amide I band (C = O stretching vibration) in the region 1,600–1,700 cm^−1^ and the amide II band 1,520–1,580 cm^−1^ (Figure 4). Both amide I and amide II bands were identified in different formulations of cationic and anionic liposomes confirming that the coating with WPI had been achieved. Figures 4, 5 clarify that amide bands of WPI were pronounced when WPI was the second coating layer of the liposomes after the pectin. Thus, WPI had preferential interactions with the pectin compared to liposomes. These results suggest other interactions additional to the electrostatic interaction occur between the WPI and pectin. It is possible that physical entanglement between the polymer chains and the protein is an important factor in the coating of liposomes by the WPI. This phenomenon is already known in the interaction of pectin and mucin and explains the muco-adhesive properties of the polymer (Takeuchi et al., 2005; Klemetsrud et al., 2013).

Pectin has a fingerprinting area in the range 1,400 and 800 cm^−1^ (Winning et al., 2009) with a distinctive peak in the amide region at 1,700 cm^−1^ confirming the amidation of the pectin as indicated previously. Pectin bands were identified in all liposomes coated with pectin confirming the effectiveness of pectin coating. Strong CH_2_ peaks characterized the anionic liposomes after coated with pectin (Figure 5A) indicating that coating of negatively charged liposomes with pectin implies other types of interaction that are not electrostatic interactions. These interactions have been previously explained as the formation of hydrogen bonds between pectin and phospholipids (Zhou et al., 2014). These results confirm the results found by the zeta potential and the size analysis.

### Morphological analysis by TEM

**Figure 6:**
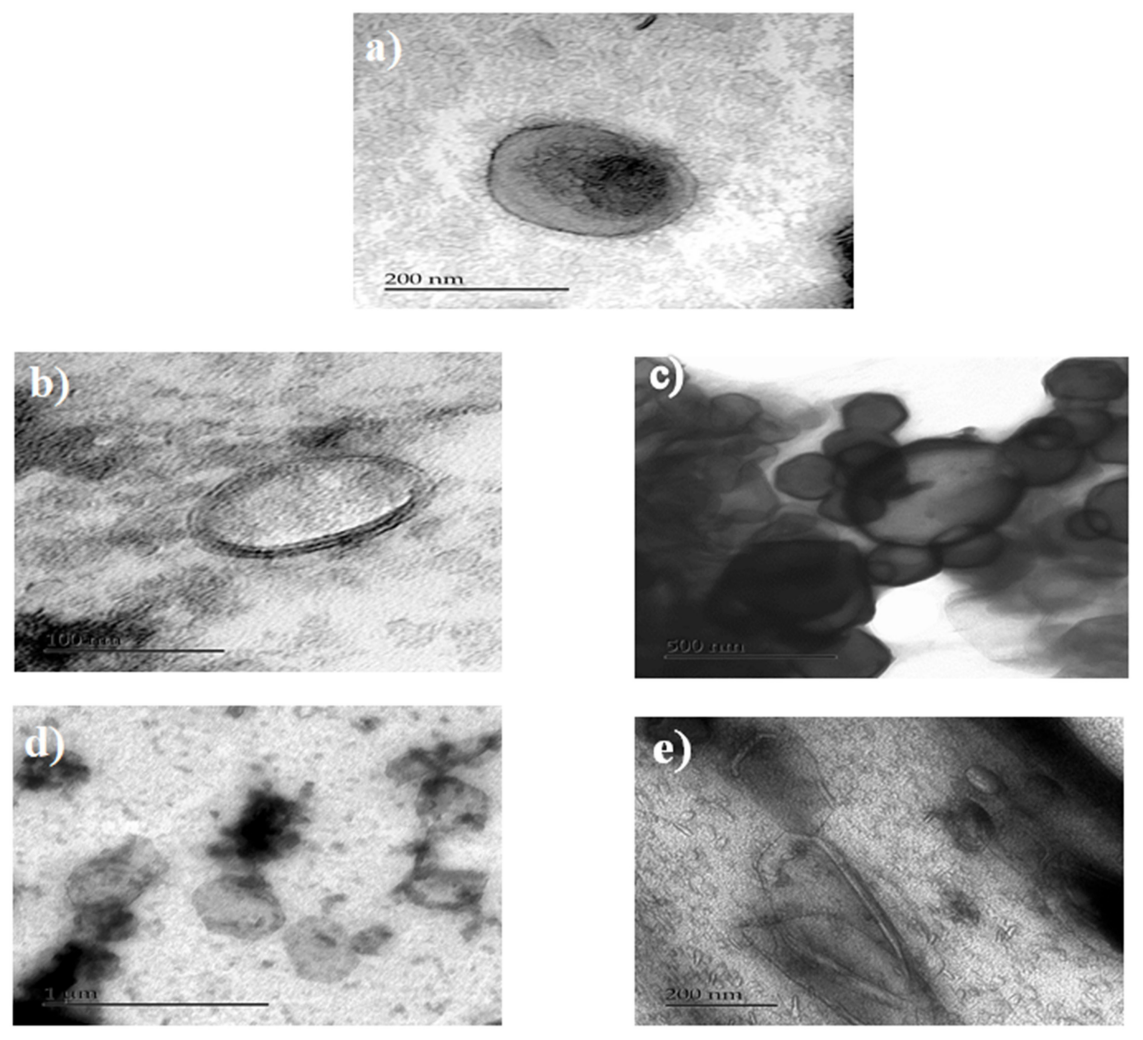
TEM of anionic liposomes, **(a)** non-coated, **(b)** WPI-coated liposomes, **(c)** pectin-coated liposomes, **(d)** pectin/WPI coated liposomes, and **(e)** WPI/ pectin coated liposomes.

We selected the anionic liposomes for TEM studies. TEM images of anionic liposomes are presented in Figure 6. Analyses by TEM of anionic liposomes clarified the differences between the different formulations. Regarding the formulations of anionic liposomes, an overall increase in size of the liposomes was observed after coating which confirmed the results from the analysis of the particle size. In addition, the analysis in TEM allowed us to confirm the coating of anionic liposomes by pectin and / or WPI. TEM images showed that non-coated liposome formulations were mainly unilamellar liposomes. However, a few multi-lamellar type liposomes were found with a spherical shape. Unilamellar liposomes are the consequence of the sonication step during the preparation of liposomes. In addition, the TEM analysis enabled confirmation that the coating of anionic liposomes by a pectin layer was likely due to hydrogen bonds (Zhou et al., 2014). Pectin layer was shown in the microscopy images as a very smooth black layer. The images showed that pectin helped to maintain the shape and integrity of the liposomes (Figure 6c). Unlike pectin, WPI did not form an external layer on the liposomes. Alternatively, the TEM images of WPI showed a diffused appearance without maintaining the integrity of the liposomes thus indicating that WPI was inserted into the membrane. The results are in agreement with (Frenzel and Steffen-Heins, 2015) who reported that under acidic conditions WPI is predominantly in a molten globule state and could be inserted into the membrane (Figure 6b). TEM images showed two major differences between the double coated formulations in the shape and the appearance of the liposomes. (Figures 6d,e). In addition, it also confirmed that the second coating layer has been achieved. When the anionic liposomes were coated with WPI followed by pectin, the form of vesicles was much more elongated as compared to the case of coating with pectin followed by WPI where the liposomes preserved their original shape. This explains the decrease in size and the compression effect when liposomes are coated with the WPI, as shown in size and zeta potential data. Moreover, when the external coating layer consisted of WPI, liposomes had a diffuse appearance. Differences in liposome surface appearance when coated with whey or pectin could be explained by the difference in molecular weight of the two coating agents. Indeed, pectin is a branched anionic polysaccharide whose molecular weight may vary between 50 and 150 kDa while WPI comprises a mixture of proteins whose main protein is β-lactoglobulin which is 18 kDa. This difference in surface appearance may suggest differences in the types of interactions that take place between pectin, WPI and the liposome membrane and reinforces the results and assumptions found with the F-IR analysis of our formulations.

### Encapsulation efficiency (EE%)

The double coating of liposomes required optimization to improve the EE%. Indeed, the levels of MccJ25 after coating with two layers were non-detectable by HPLC. Accordingly, during the production of liposomes, different steps were implemented to improve the encapsulation efficiency and also the handling steps of liposomes were reduced to minimize losses of encapsulated MccJ25. All coating steps and centrifugations were performed at 4°C to remain below the *T*_m_ of phospholipids (24°C). Moreover, phospholipids concentration was optimized to 6 mM and the lipid / active ingredient (MccJ25) ratio was optimized to 4:1, respectively. Finally, similar concentrations of MccJ25 were added to the coating solution to balance or counter the passive diffusion of the microcin during coating. The degree of encapsulation obtained using these optimization steps was significantly higher for the formulations with a dual coating. Table 2 presents the EE% of different coated and non-coated liposomes. The EE% was comparable to that reported in the literature (da Silva Malheiros et al., 2010). During the extrusion step, some of the active ingredient was diffused and lost. This result can be explained by the fact during extrusion the lipid bilayers are in critical conditions as they are subject to great pressure and are forced to move through a polycarbonate membrane with pore size of 300 nm. Therefore, the remaining amount of MccJ25 was measured after the extrusion. The quantities of MccJ25 were similar to the differences in the EE% before and after extrusion. These results are consistent with those previously reported when encapsulating a lipopeptide in liposomes (Liang et al., 2005). In addition, the diffusion of MccJ25 during extrusion was greater in negatively charged liposomes as compared with the positively charged liposomes (−3.6 and −2.2%, respectively). This can be explained by the extrusion effect on increasing the negatively charged surface of liposomes and hence the electrostatic repulsion between the anionic phospholipids and MccJ25 which is negatively charged at pH 7. Table 2 shows that the encapsulation rates decrease with the number of coating layers. One possible explanation could be a loss of some liposomes after each centrifugation step in the supernatant. Another hypothesis could be the interaction between the MccJ25 and materials used for the coating. Indeed, as the liposomes are associated with pectin and/or WPI, characteristic peaks of coatings matrices appeared during analysis by HPLC. To confirm this hypothesis, all these fractions were collected and tested for their antimicrobial activity against *S. enteritidis* with the agar diffusion test. No activity was found confirming that MccJ25 does not interact with WPI or pectin.

**Table 2:** Encapsulation efficiency of different liposomal formulas.

**Liposome**	**Anionic**	**Cationic**
Non-coated liposomes (before extrusion)	23.69 ± 0.55^a^	26.82 ± 1.23^a^
Non-coated liposomes (after extrusion)	20.09 ± 0.37^b^	24.62 ± 1.41^a^
Liposome + pectin	10.26 ± 0.06^c^	4.68 ± 0.20^d,e^
Liposome + WPI	9.60 ± 0.27^c^	4.32 ± 0.29^d,e^
Liposome + pectin + WPI	7.60 ± 0.52^c,d^	2.86 ± 0.28^f^
Liposome + WPI + Pectin	5.70 ± 0.47^d^	1.56 ± 0.18^f^

*Encapsulation efficiency was measured by HPLC and expressed as % of encapsulated MccJ25 before and after encapsulation. Results are expressed as average ± standard deviation. Different letters express significant differences at each sampling time*.

The cationic liposomes showed a significantly lower degree of encapsulation efficiency after coating compared with anionic liposomes. One possible explanation could be that the MccJ25 interacts and encapsulates mainly in the lipid bilayer of the cationic liposomes. Therefore, during the coating steps, a competition occurred between MccJ25 and the more electrostatic coating agents, resulting in the release of MccJ25. Additionally, encapsulation efficiency was significantly lower when WPI constituted the first coating layer. This decrease in encapsulation may be due to the large pressing phenomenon exerted on the WPI unshaped liposomes as has been discussed in previous sections. Conversely, the degree of encapsulation of liposomes after coating with pectin as a first layer was higher. It has been reported that pectin does not interact with the interior of the double membrane of the liposomes which therefore decreases the loss of encapsulated active ingredient (Zhou et al., 2014). This is in agreement with the results found by TEM, where pectin appeared to maintain the integrity of the membrane whereas the WPI the interacted with the lipid membrane. Finally, encapsulated MccJ25 presented a strong antimicrobial activity at the reported EE%.

### Dissolution model for *in Vitro* simulated digestion

**Figure 7:**
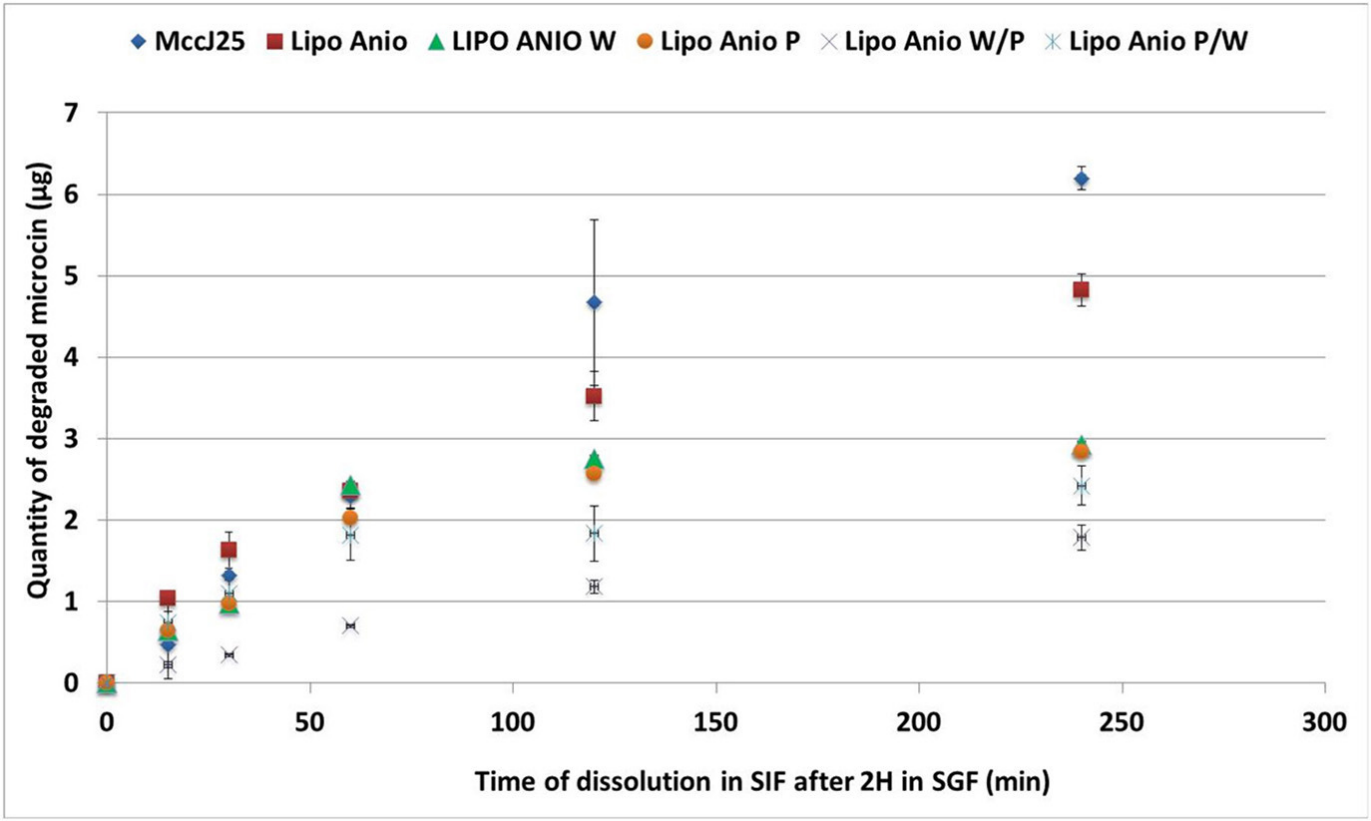
Dissolution of encapsulated MccJ25 in simulated intestinal conditions for 4 h after 2 h of digestion in simulated gastric conditions. Lipo Anio, anionic liposomes; Lipo Anio W, anionic liposomes coated with whey; Lipo Anio P, anionic liposomes coated with pectin; Lipo Anio W/P, anionic liposomes coated with whey and subsequently coated with pectin; Lipo Anio P/W, anionic liposomes coated with pectin and subsequently coated with whey. Error bars indicate the standard deviation of two measurements.

The optimum concentration of enzymes to be used for the digestion of the encapsulated MccJ25 intestinal phase was determined using a preliminary test. The degraded amount of MccJ25 after 4 h digestion in SIF was below 50%, which showed that the peptide is fairly resistant to enzyme hydrolysis. This result seems consistent given the specific lasso structure of this bacteriocin and is consistent with other studies which established that its structure provides protection against enzymatic lysis (Blond et al., 1999). MccJ25 resisted gastric digestion in all liposomal formulations. Such resistance was expected as there is no preferential cleavage site of pepsin on the peptide primary structure (Ehren et al., 2008). However, theoretical cleavage sites were found for chymotrypsin (Gráf et al., 2013) and elastase (de Oliveira and Salgado, 2013). During the intestinal dissolution phase, the degradation of MccJ25 was significantly different depending on the formulation (Figure 7). Liposomes provided protection to the MccJ25 during digestion in the intestinal phase as the amount of degraded MccJ25 after 4 h SIF were less than the degraded amount of free microcin. Anionic liposomes were more efficient in the protection of MccJ25 as compared with cationic liposomes. One layer coated liposomes provided interim protection to the MccJ25 compared to non-coated formulations. Double coated liposomes with WPI and pectin offered the best protection to the peptide during *in vitro* digestion where the protection was significantly higher than other formulations after 30 min of digestion and until the end of the digestion. The second double coated formulation (pectin / WPI) showed a significant lower degraded amount of MccJ25 than that obtained with single coated liposomes or non-coated liposomes after 2 h digestion. Thus, we can conclude that the double coating provided protection to the microcin throughout the gastrointestinal tract. Accordingly, the colon would be the target site of action. Previous studies have established that a single layer of pectin or WPI improves the stability of liposomes (Ehren et al., 2008; Frenzel and Steffen-Heins, 2015; Frenzel et al., 2015).

## Conclusion

In this study, microsin J25, a potent antimicrobial peptide, was encapsulated in both positively and negatively charged liposome that were subsequently coated with pectin and/or WPI. The coating process was optimized to improve the EE% and the protection of microcin against gastrointestinal digestion. Our results highlight the potential use of liposome coating technology as a suitable carrier for protecting bacteriocins from gastrointestinal enzymes. The dual coating liposomes provided additional protection to the microcin during simulated gastrointestinal digestion. Further studies to explore the biological muco-adhesion properties are required. It is also important to consider the use of more affordable sources of phospholipids such as lecithin for large-scale commercial production of liposomes.

## Author contributions

AG: participated in the lab work, supervising lab work, interpretation of data, drafting the manuscript, performing the statistical analysis. CM: participated in the lab work, interpretation of data. RH: design of the work, interpretation of data. IF: supervised the research, final approval of the version to be published. MS: supervised the research, final approval of the version to be published.

### Conflict of interest statement

The authors declare that the research was conducted in the absence of any commercial or financial relationships that could be construed as a potential conflict of interest.

## References

[B1] Aguilar K. C. Tello F. Bierhalz A. C. Romo M. G. G. Flores H. E. M. Grosso C. R. (2015). Protein adsorption onto alginate-pectin microparticles and films produced by ionic gelation. J. Food Eng. 154, 17–24. 10.1016/j.jfoodeng.2014.12.020

[B2] Blond A. Péduzzi J. Goulard C. Chiuchiolo M. J. Barthélémy M. Prigent Y.. (1999). The cyclic structure of microcin J25, a 21-residue peptide antibiotic from *Escherichia coli*. FEBS J. 259, 747–756. 10.1046/j.1432-1327.1999.00085.x 10092860

[B3] da Silva Malheiros P. Daroit D. J. Brandelli A. (2010). Food applications of liposome-encapsulated antimicrobial peptides. Trends Food Sci. Technol. 21, 284–292. 10.1016/j.tifs.2010.03.003

[B4] de Oliveira E. B. Salgado M. C. O. (2013). Pancreatic elastases. Handb. Proteolytic Enzymes 3, 2639–2645. 10.1016/B978-0-12-382219-2.00584-6

[B5] Ehren J. Govindarajan S. Morón B. Minshull J. Khosla C. (2008). Protein engineering of improved prolyl endopeptidases for celiac sprue therapy. Protein Eng. Design Sel. 21, 699–707. 10.1093/protein/gzn050 18836204 PMC2583057

[B6] Ennaas N. Hammami R. Gomaa A. Bédard F. Biron É. Subirade M.. (2016). Collagencin, an antibacterial peptide from fish collagen: activity, structure and interaction dynamics with membrane. Biochem. Biophys. Res. Commun. 473, 642–647. 10.1016/j.bbrc.2016.03.121 27038545

[B7] Frenzel M. Steffen-Heins A. (2015). Whey protein coating increases bilayer rigidity and stability of liposomes in food-like matrices. Food Chem. 173, 1090–1099. 10.1016/j.foodchem.2014.10.076 25466129

[B8] Frenzel M. Krolak E. Wagner A. Steffen-Heins A. (2015). Physicochemical properties of WPI coated liposomes serving as stable transporters in a real food matrix. LWT-Food Sci. Technol. 63, 527–534. 10.1016/j.lwt.2015.03.055

[B9] Gomaa A. I. Sedman J. Ismail A. A. (2013). An investigation of the effect of microwave treatment on the structure and unfolding pathways of β-lactoglobulin using FTIR spectroscopy with the application of two-dimensional correlation spectroscopy (2D-COS). Vib. Spectrosc. 65, 101–109. 10.1016/j.vibspec.2012.11.019

[B10] Gráf L. A. Szilágyi L. A. Venekei I. A. (2013). Chymotrypsin, in Handbook of Proteolytic Enzymes Vol. 3 (Elsevier Ltd.), 2626–2633. 10.1016/B978-0-12-382219-2.00582-2

[B11] Hammami R. Bédard F. Gomaa A. Subirade M. Biron E. Fliss I. (2015). Lasso-inspired peptides with distinct antibacterial mechanisms. Amino Acids 47, 417–428. 10.1007/s00726-014-1877-x 25466905

[B12] Hammami R. Zouhir A. Hamida J. B. Neffati M. Vergoten G. Naghmouchi K. (2009). Antimicrobial properties of aqueous extracts from three medicinal plants growing wild in arid regions of Tunisia. Pharm. Biol. 47, 452–457. 10.1080/13880200902822604

[B13] Jesorka A. Orwar O. (2008). Liposomes: technologies and analytical applications. Annu. Rev. Anal. Chem. 1, 801–832. 10.1146/annurev.anchem.1.031207.112747 20636098

[B14] Klemetsrud T. Jonassen H. Hiorth M. Kjøniksen A.-L. Smistad G. (2013). Studies on pectin-coated liposomes and their interaction with mucin. Colloids Surf. B 103, 158–165. 10.1016/j.colsurfb.2012.10.012 23201733

[B15] Lähdesmäki K. Ollila O. H. Koivuniemi A. Kovanen P. T. Hyvönen M. T. (2010). Membrane simulations mimicking acidic pH reveal increased thickness and negative curvature in a bilayer consisting of lysophosphatidylcholines and free fatty acids. Biochim. Biophys. Acta 1798, 938–946. 10.1016/j.bbamem.2010.01.020 20132791

[B16] Lasic D. D. Papahadjopoulos D. (1995). Liposomes revisited. Science 267:1275. 10.1126/science.7871422 7871422

[B17] Liang M. T. Davies N. M. Toth I. (2005). Encapsulation of lipopeptides within liposomes: effect of number of lipid chains, chain length and method of liposome preparation. Int. J. Pharm. 301, 247–254. 10.1016/j.ijpharm.2005.06.010 16054787

[B18] Liu W. Liu J. Liu W. Li T. Liu C. (2013). Improved physical and *in vitro* digestion stability of a polyelectrolyte delivery system based on layer-by-layer self-assembly alginate–chitosan-coated nanoliposomes. J. Agric. Food Chem. 61, 4133–4144. 10.1021/jf305329n 23566223

[B19] Liu W. Ye A. Liu C. Liu W. Singh H. (2012). Structure and integrity of liposomes prepared from milk-or soybean-derived phospholipids during *in vitro* digestion. Food Res. Int. 48, 499–506. 10.1016/j.foodres.2012.04.017

[B20] Liu W. Ye A. Liu W. Liu C. Han J. Singh H. (2015). Behaviour of liposomes loaded with bovine serum albumin during *in vitro* digestion. Food Chem. 175, 16–24. 10.1016/j.foodchem.2014.11.108 25577045

[B21] Lowry O. H. Rosebrough N. J. Farr A. L. Randall R. J. (1951). Protein measurement with the Folin phenol reagent. J. Biol. Chem. 193, 265–275. 14907713

[B22] Millette M. Le Tien C. Smoragiewicz W. Lacroix M. (2007). Inhibition of *Staphylococcus aureus* on beef by nisin-containing modified alginate films and beads. Food Control 18, 878–884. 10.1016/j.foodcont.2006.05.003

[B23] Narsaiah K. Jha S. Wilson R. A. Mandge H. Manikantan M. Malik R. (2013). Pediocin-loaded nanoliposomes and hybrid alginate–nanoliposome delivery systems for slow release of pediocin. Bionanoscience 3, 37–42. 10.1007/s12668-012-0069-y

[B24] Nguyen S. Alund S. J. Hiorth M. Kjøniksen A.-L. Smistad G. (2011). Studies on pectin coating of liposomes for drug delivery. Colloids Surf. B 88, 664–673. 10.1016/j.colsurfb.2011.07.058 21862293

[B25] Sable S. Pons A.-M. Gendron-Gaillard S. Cottenceau G. (2000). Antibacterial activity evaluation of microcin J25 against diarrheagenic *Escherichia coli*. Appl. Environ. Microbiol. 66, 4595–4597. 10.1128/AEM.66.10.4595-4597.2000 11010926 PMC92352

[B26] Souza F. N. Gebara C. Ribeiro M. C. Chaves K. S. Gigante M. L. Grosso C. R. (2012). Production and characterization of microparticles containing pectin and whey proteins. Food Res. Int. 49, 560–566. 10.1016/j.foodres.2012.07.041

[B27] Takeuchi H. Thongborisute J. Matsui Y. Sugihara H. Yamamoto H. Kawashima Y. (2005). Novel mucoadhesion tests for polymers and polymer-coated particles to design optimal mucoadhesive drug delivery systems. Adv. Drug Deliv. Rev. 57, 1583–1594. 10.1016/j.addr.2005.07.008 16169120

[B28] Winning H. Viereck N. Salomonsen T. Larsen J. Engelsen S. B. (2009). Quantification of blockiness in pectins—a comparative study using vibrational spectroscopy and chemometrics. Carbohydr. Res. 344, 1833–1841. 10.1016/j.carres.2008.10.015 19101665

[B29] Zhou W. Liu W. Zou L. Liu W. Liu C. Liang R.. (2014). Storage stability and skin permeation of vitamin C liposomes improved by pectin coating. Colloids Surf. B 117, 330–337. 10.1016/j.colsurfb.2014.02.036 24681045

